# Struggling with memory: anguish and hope in selected pandemic poems by migrant workers in Singapore

**DOI:** 10.3389/fsoc.2024.1501520

**Published:** 2024-12-31

**Authors:** Noel Christian A. Moratilla

**Affiliations:** University of the Philippines Diliman, Quezon City, Philippines

**Keywords:** pandemic poetry, migration, labor export, domestic work, COVID

## Abstract

The pandemic has tested the fortitude and resilience of a huge swath of humanity. Even measures undertaken to address the pandemic, primarily the massive vaccination campaigns, revealed a glaring disparity between and within societies. The collective grief, anxiety, and desire for survival have led to creative ways to contend with the crisis. Poetry has served as one of those strategies. This paper revolves around selected pieces of poetry particularly those with themes related to being an Asian migrant worker during the pandemic. The primary themes drawn from the poems are as follows: dealing with the changes brought about by the pandemic; concern over family; job precarity and stigmatization; and hope and resilience.

## 1 Introduction

It is no exaggeration that the COVID 19 pandemic may be considered as the first serious global health emergency of the 21st century, having wrought incalculable damage especially in its first few years, and continues to do so albeit to a much less serious degree. Asia is one of those badly hit by the dreaded disease with millions of reported deaths as borne out by the following figures: India with more than 530,000, Indonesia with more than 160,000, Japan with more than 74,000, and the Philippines with more than 66,000 (Statista, 2024). Add to these figures the numbers of people who contracted the virus—more than 7 billion worldwide according to reports. The impact on the economy was also unsettling, with hundreds of thousands of companies closing shop if not temporarily halting their operations especially in the first few months of the global emergency, thus leading to the displacement of millions of workers with families to feed. It is not surprising, therefore, that the number of impoverished people likewise increased exponentially. In 2021 article published by the World Bank no less, it was revealed that “the average incomes of people in the bottom 40% of the global income distribution (were) 6.7% lower than pre-pandemic projections” (Hill et al., 2021; p. 1).

Migrant workers are among the sectors that reeled from the pandemic and its adverse repercussions not only health-wise but also economically and politically. It is this class of people—or what I would call the “globaletariat”—who exert their energy in order to sustain the global, neoliberal order, but whose welfare is far from guaranteed and whose most fundamental rights are coldly ignored if not brazenly violated. The inequality that characterizes the global economy became even more demonstrable during the confusion and disturbance engendered by the medical crisis. Before the pandemic, there were serious concerns about the welfare of overseas workers, including their employment security and physical safety. With the onslaught of the COVID-19, migrant workers found themselves in circumstances that were even worse than what they had been experiencing prior to the pandemic. Job precarity became more apparent with the compulsory return of migrants to their country of origin. According to the International Labor Organization, migrants were repatriated “without operational systems and protocols in place to ensure safe repatriation or to share the cost burden of return trips and quarantine between countries of origin, countries of destination and employers” (Jones et al., 2021, p. ix). Given the difficulty of reintegration, workers found themselves longing to migrate again to continue providing for their families. Out of desperation, some have even resorted to seeking the assistance of unscrupulous agents and end up getting trafficked. The United Nations Office on Drugs Crime (2021) stated, “During the COVID-19 pandemic, irregular migrants (including undocumented and/or unaccompanied children and women), who often use the services of smugglers during their journey, have been among the most exposed to trafficking in persons, as their vulnerabilities have been exacerbated by restrictions to limit the spread of the virus” (p. 12).

Workers who did not get repatriated immediately were cast into dehumanizing conditions, with some of them getting quarantined in overcrowded, unhygienic facilities, thus making them even more susceptible to the virus. In April 2020, just a few weeks after the lockdowns in many parts of Asia, Amnesty International (2020) called for more humanitarian measures in placing workers under strict quarantine: “Quarantines must always be imposed in a manner consistent with human rights… (it should be ensured) that human rights remain central to all attempts at prevention and containment of the COVID-19 virus, and that all people have access to adequate space for social distancing, adequate water and sanitation facilities, and access to proper healthcare and hygiene equipment for everyone affected, without discrimination” (para. 4).

## 2 The “globaletariat,” Singapore's migrants, and pandemic poetry

As has been stated, the economic system of the contemporary globalized world is ostensibly neoliberal in orientation and in practice. Neoliberalism is characterized by, among other things, the prioritization of private interests leading to the deregulation of markets and the privatization of public assets. Under these conditions, governments also abandon the responsibility to provide employment opportunities and pass it on to private hands. In consequence, workers may find themselves working outside their homeland, expending their energy and using their skills in favor of a country not their own. At a certain point, this will prove detrimental to the welfare of one's country of origin which, because of the massive migration of its workers, will have to deal with shortages in its own labor force. As pointed out by Kotz (2002), neoliberalism has resulted in, among other things, the “elimination of, or cutbacks in, social welfare programs” (p. 65). Thus, the impoverished class of society is the one that is most susceptible to the lure of labor migration, leading to what Delgado Wise (2018) called “forced displacement” (p. 14). In many instances, even “restrictive” policies relative to migration cannot discourage potential migrants from entering a country and working there, exemplifying the contradictions embedded in what is called “gap hypothesis” (Helms and Lebland, 2019, p. 7).

It is, therefore, naïve to consider neoliberalism as nothing but a paradigm of contemporary development adapting to the conditions of global economic order; rather, it is a vicious economic and political philosophy that can widen further the gap between the rich and the poor. Saad-Filho and Johnson (2007 as cited in Moratilla, 2019) described neoliberalism as a “beast” that only brings ruin and largely “trespasses into new territories, tramples upon the poor, undermines rights and entitlements, and defeats resistance” (p. 5). Such a critical assessment also resonates with that of Delgado-Wise (2014) who characterized neoliberalism as a main factor for “deepening” asymmetries—first, those “within countries and between countries and regions”; and second, social inequalities that have further contributed to poverty, exploitation, discrimination, and the “sharp decline in living and conditions” (p. 651).

Under the neoliberal system, migrant workers have to grapple with the same, chronic concerns besetting workers in general including abuse and job precarity. The only difference is that they struggle with these issues while away from the homeland. Even the World Bank (2023)—invariably recognized as a chief implementor of neoliberalism—acknowledges that labor migration is by no means a guarantee against inequality. Even the sending of remittances is inextricable from the issue of class because “wealthier households can more easily afford to send migrants abroad and thus earn higher remittances” (World Bank, 2023, p. 131). Migrants within and from Southeast Asia have been cited among the most vulnerable migrant groups. As per the Business Human Rights Resource Center (2023), Asia was the setting of about 30% of abuses against migrant workers in 2023 although the figures could be higher because of “restrictions on journalistic freedoms, lack of access to remedy or grievance mechanisms by migrant workers and the threat of reprisal for workers” (What have we discovered? para. 3). The prevalence of abuses involving Southeast Asian migrants could be partly ascribed to the nature of the work performed by the victims who were mostly domestic helpers or sex workers.

It is no exaggeration that migration is closely linked with the economic disparity between nations. Workers leave their homeland to look for financial security especially in more economically advanced countries. In Southeast Asia, economic superpower Singapore has been a favorite destination of workers from various countries. According to Singapore Ministry of Manpower (2023), the branch of government primarily concerned with the workforce in the island country, there were more than 1.4 million foreign workers in 2019. The number saw a decline in the years during the pandemic, but in 2023, with the easing of restrictions on foreign workers, the number rose to more than 1.5 million, of whom about 20 percent were domestic workers (https://www.mom.gov.sg/foreign-workforce-numbers).

However, migration is not without its share of issues and concerns because in many parts of the world, migrants have to navigate through strict migration and work policies, not to mention systemic racism and other forms of injustice. While there have been efforts to uphold the rights of migrants in keeping with the ideals of equality and inclusion, they are still largely deprived of the rights exercised by natives and citizens. For example, Singapore has no policy on minimum salary for work permit holders, and because foreign workers have to secure work permits from the government, there are no salary standards for them, only salary guidelines (The Independent Singapore, 2023). Years before the pandemic, concerns over violations of migrant workers' rights in Singapore had already been rife. Jolovam Wham, acting executive director of Singapore's Humanitarian Organisation for Migration Economics, noted that abuses, exploitative recruitment fees, and non-payment or underpayment of wages were among the problems confronting migrant workers in the country (Bacani, 2017).

The challenges plaguing migrant workers, including their “disproportionate” treatment, became more complicated during the pandemic (Migration Data Portal, 2023). Singapore, despite its reputation as a largely benevolent host country to migrants, may be cited as a classic case. In a December 2020 report, it was claimed that within 9 months, almost 50% of Singapore's migrant workers had been infected with COVID, and this was partly due to the strict implementation of the lockdowns. Speaking on behalf of Transient Workers Count Too (TWC2), a non-government organization, Alex Au asserted, “The figures don't surprise us… During the middle of the year, workers who tested positive were telling us that they were told to stay in their rooms and not taken into isolation. They remained in contact with their roommates” (Illmer, 2020). The restrictions supposedly targeting migrant workers continued even after a largely vaccination campaign had started, thus affecting the workers' wellbeing and leading to an increase in suicide rates. According to Professor Jeremy Lim of the National University of Singapore (NUS), the lockdowns weighed on the migrant workers' mental health: “From a public health point, there is no danger of letting the workers out of their dorms” (Jin, 2021). The situation is so serious that complete racial harmony and social cohesion may just be aspirational for now. As Steinhauer (2022) observed, “This state-sponsored abuse not only disregards the human rights of migrant workers but also fuels an undercurrent of stigma and xenophobia that undermines any attempt by the Singapore government to achieve racial harmony” (para. 1).

The collective grief, anxiety, and desire for survival have led to creative ways to contend with the destruction brought about by the disease and its profound impact. Some visual artists, for instance, drew inspiration from the heroic sacrifices of medical professionals. Others have turned to the writing of fiction and non-fiction books revolving around the pandemic. Poetry has likewise served as a strategy for memorializing the lived experiences of despair and hope during the pandemic, including their imbrication with issues of justice, equality, empowerment, and representation.

In the discussion, I shall be dealing with selected pieces of poetry, particularly those with themes related to being an Asian migrant worker during the pandemic. I shall be showing how during the pandemic, poetry has served as a discursive tool on the part of these workers to navigate their experiences, exercise individual and collective agency, and resist excesses of power. Specifically, the analysis will be guided by the notion of “public time” as defined by Henry Giroux (2021) within the context of a global crisis necessitating not just political or medical solutions, but also the pedagogical practices and opportunities for resistance and the emergence of critical discourses. The following explanation of the said concept is instructive: “Rather than maintaining a passive attitude toward power, public time demands and encourages forms of political agency based on a passion for self-governing, actions informed by critical judgment, and a passion for linking responsibility and social transformation. Public time renders governmental power explicit, and in doing so it rejects the language of religious rituals and the abrogation of the conditions necessary for the assumption of basic freedoms and rights” (Giroux, 2011, p. 549).

The notion of public time as explicated by Giroux (2003) is one that challenges the connection of time to economic productivity and consumerism by pointing out its political character and its alternative use for “consideration, contemplation, and critical thinking” (p. 148). Public time, in other words, can be appreciated by viewing it as a different kind of rationality that is critical of institutions of power while at the same time acknowledging democratic possibilities through the recuperation of various narratives and voices.

Literature serves as one way by which the responses of people to the pandemic, whether superficial or profound, are verbalized and memorialized. This strategy can help us comprehend our reactions and the “narratives that surround the spread of the coronavirus” such as those that perpetuate social and cultural inequalities—“racism, xenophobia, ableism” (Haith, 2020). Literature—or creative writing—can thus be imbued with a critical orientation against the excess of power that reinforces various forms of injustice. In this case, critical reflexivity is a necessary component of the writing in order to “process the upheavals happening in our world and ourselves” (Mayor and Pollack, 2022, p. 383). Writing here is not just cerebral or cognitive but “emotional and embodied”—a veritable strategy of “connecting with our emotions, body, and spirit/imagination… to distill our complex experiences” especially in times of crisis (p. 386). What is important to note is that creative writing cannot just be reduced to a strategy of self-expression; rather, it constitutes a way by which the writer and, necessarily, the reader, take cognizance of their position within the context of “harmful ideologies and structures” through “emotions, body, and spirit” (p. 392). In appraising the importance of literature, writing, and even other creative arts during the pandemic, one can also invoke the Foucauldian concept of “technology of the self” as a technique of maintaining coherence, stability, and self-care. In the words of Foucault (1988) himself, technologies of the self allow individuals to “effect by their own means or with the help of others a certain number of operations on their own bodies and souls, thoughts, conduct, and way of being, so as to transform themselves in order to attain a certain state of happiness, purity, wisdom, perfection, or immortality” (p. 18). By this, Foucault referred to the strategies through which individuals act upon themselves without deemphasizing the realities of power and domination. In this paper, I will try to demonstrate how the migrant workers, despite their conditions and the difficulties imposed on them by the health crisis, used such strategies through poetry.

## 3 The materials

While the previous section established the practice of producing literature as a strategy of coping with the pandemic, there has been a dearth of materials pertinent to the subject of this paper. It is because of at least two things: First, there have been few publications by migrant workers, particularly those belonging to relatively low-income groups. Second, poetry is not prevalent among migrant workers who do write or have been published, partly because of the inherent features of the genre—the tautness of the language, the use of images and rhetorical devices, the specialized vocabulary. There are arguably more materials belonging to the other traditional genres, say, the essay and the short fiction. In any case, the materials were culled from four sources that are based in or associated with Singapore, three of which are online and one printed.

The sources include Kopi, a blogsite which declares itself to be dedicated to making Singaporeans “discover their inner workings of their country”—its language, its politics, its culture, its people.” The essay, “Poetry From Migrant Workers Shines a Light on Their COVID-19 Plight,” from which the poems were selected was dedicated to the “growing migrant literary scene” in the face of the “coronavirus outbreaks in the migrant communities” (On the importance of narrative and literature right now, para. 4).

The second source is an online version of Art Review, magazine established in 1949, which prides itself on being “one of the world's leading international contemporary art magazines, dedicated to expanding contemporary art's audience and reach, and tracing the ways it interacts with culture in general.” Admirably, some of the issues are devoted to the writings of migrants. The specific issue from which the materials for this study was drawn made the following observation: “In recent months, mass outbreaks in cramped migrant worker dormitories have forced the city-state into lockdown, renewing debate and scrutiny around the poor treatment of such workers” (Khokan, 2020).

The third source, also online, is the Singapore Red Cross website which published pdf versions of submissions that won in the 2020 poetry writing competition called “Words Heal the Mind”; the said competition was organized by the Singapore Migrant Writers group. As its promotional material declared, the competition aimed to raise “awareness of the importance of mental health especially among migrants” at a time of considerable uncertainty because of the pandemic (Creative minds commemorate world mental health day, 2021).

The last source is the printed book *Call and Response 2: A Singapore Migrant Anthology* published by Math Paper Press and edited by Khokan et al. (2021). In the “Afterword,” Poh Yong Han describes the book as an anthology of the writings of the migrant workers in Singapore—a “node in the greater migrant ecosystem, one that is incredibly transnational and dynamic” (p. 304). Moreover, the collection is designed to foster “more migrant-local dialogue” and celebrate “migrant voices” (p. 304).

It should also be noted that most of the migrant workers whose works are cited in this study are engaged in skilled labor and, in that regard, do not have the educational and cultural trappings of published expatriate writers based primarily in the West. They are purposely selected because they are reckoned to be the underrepresented sector in the world's migrant population and, thus, deserve to be accorded due recognition in research projects concerning diasporic literature.

## 4 Analysis and discussion

From the poems selected and studied for the paper, I was able to tease out three key themes: a world changed by the pandemic; concern over the family; job precarity and stigmatization; and hope despite the sense of uncertainty brought about by the pandemic. Note also how the pieces show reflexivity on the part of their writers since many of the lived experiences cited resonate very well with actual experiences especially during the first few months of the pandemic when lockdowns were in effect, and many had to deal with the actual or potential loss of employment. It is not surprising why many of the sources analyzed here reference the travails of the migrant worker during the crisis.

### 4.1 Changed world

It is not surprising that the feelings of fear, perplexity, and restiveness can be gleaned from many of the poems. These feelings are brought about by how the world has changed since the onset of the COVID-19 emergency which, as pointed out by Butler (2021) foregrounds the “fact that we are implicated in a shared world” (para. 1). With the pandemic and its serious implications, the world transitioned dramatically into something fraught with uncertainty. In “Singapore Circuit Breaker: 29th Day” by MD Sharif Udin (Kopi blogsite, 2020), for instance, the persona laments her/his inability to perform many of the quotidian activities that s/he used to enjoy—“I wish to sit in the front seat on the top deck of/bus number 67/and walk around the narrow streets of the city then buy a three dollar big/sweep ticket.” Conveyed here is the desire to experience anew a life of normalcy that COVID 19 has apparently derailed.

The same feelings can be drawn from the poem “Pandemic” by domestic helper Warminingsih (Kopi blogsite, 2020). Here, the persona expresses her/his belief “that it is just a scary dream stuff/from a movie scene” as if trying to allay her fears. The poem likewise describes how the situation has led to apocalyptic repercussions through images that filled with dread the first few months of the crisis—“circuit breaker lockdowns,” “panic buying” that “stripped the shelves bare,” and “empty streets.” The same tenor and horror are expressed in “Creating Images of Wishes of Covid-19” by domestic helper Haidee Roiles (Kopi blogsite, 2020), in which the persona recounts how she/he had to grapple with the “thunderstorm of worries and uncertainties” at a time when excessive panic was overriding any call for patience and sobriety.

The profound feelings of worry and agitation are also found in “The Pen is My Witness” by Joralyn Mounsel (Singapore Red Cross, 2021). The persona reveals a struggle with being “haunted by the situation/The present” which, given its seriousness, is an opportunity to contemplate one's own mortality, embrace it, and prepare for it. Fear is writ large in the poem but it is tempered by a recognition that the persona, once afflicted, may also end up dying like countless others: “In a minute, I might be dead, too.” Until death comes, the persona may just have to bear with the “indistinct sound/Drummed into my ears/Like the howl of the fox on a blood moon.” This was indubitably the predominant sentiment of a broad swath of humanity simply wanting to survive.

In “What is Home, When the Heart is a Nomad?” by Zasim (*Call and Response 2*, 2021, pp. 72–75), the persona begins by declaring how the feeling of happiness has become elusive and alien. Even moments of enjoyment could not alleviate the feeling of emptiness compounded by the “dark clouds” of the pandemic descending upon “our confined lives” and a sky marked by “the bloody sun,” a description that alludes to the deaths brought about by the contagion. Indeed, the days of lockdowns and isolation rendered life fragile and monotonous. Despondent, the persona cannot but wax nostalgic about the time before the pandemic when people could “wander endlessly/from one point to another.”

Unlike the poems written by skilled workers, “The End of the World in Newton” by Mrigaa Sethi (*Call and Response 2*, 2021, pp. 246–247) has a more educated middle-class sensitivity as suggested by the syntax and the images in the poem. For one, the persona in the poem is not worried about the disruption of work routines but about how once familiar sights have either changed or disappeared, particularly those that the persona used to relish during mostly solitary jogs and walks: “sunbirds (dipping) their backs in the ginger lilacs in the patio,” “the evening walkers and runners on Buckley Road,” “the bougainvillea bushes,” “the trumpet- shaped flowers littering the sidewalk,” “melodies from the piano keys.”

What is interesting about these observations is how the pandemic is viewed not just as a health emergency but as a social one to which people had to respond adequately or perish in the process. The pandemic demonstrated that the world, however vast, is inextricable from individual lives, thus necessitating a renewed stress on the collective and the communal. To quote Butler (2021), there is now a new sense of “interdependency of the world, strengthened by a common immunological predicament, challenges the notion of ourselves as isolated individuals encased in discrete bodies, bound by established borders. Who now could deny that to be a body at all is to be bound up with other living creatures, with surfaces, and the elements, including the air that belongs to no one and everyone?” (para. 5).

### 4.2 Concern over family

The fear prompted by the pandemic is not just for the persona; instead, the personae in most cases express apprehension over their family's wellbeing and understandably so. The suspension of remittance flow in the first few months of the pandemic, partly because of the loss of livelihood on the part of migrant workers, posed a considerable challenge to workers and their loved ones. The pandemic proved deleterious to the very subsistence of the families whose income largely depended on the remittance of the migrant worker (United Nations Network on Migration, 2020).

In “The Cry of a Migrant's Heart” by Udin (Kopi blogsite, 2020), the evidently male persona ruefully expresses his longing for his loved ones—his wife, his son, his siblings. Instead of their embrace, he tries to find consolation from viewing a “piece of sky bound up by the window” which, he says, “store(s) up all his troubles.” Nonetheless, there is nothing that beats being surrounded by family even—if not especially—in the middle of a crisis. The family would generally serve as a source of relief and inspiration in times of emergency especially one on a global scale. But on the part of the migrant worker, the physical distance was a formidable hurdle compounded by the restrictions imposed on travel (Cleofas et al., 2021).

Family also figures prominently in the poem “Pandemic” by Warminingsih (Kopi blogsite, 2020), wherein the poem's persona regularly checks on her/his family and exhorts them to “stay safe and healthy.” In “Covid-19” also by the same author, the persona reveals that, notwithstanding the danger posed by the pandemic on their wellbeing, they try to focus their time and energy on the work—“We are more (concerned) about our siblings' empty bellies/(Of) utmost importance is to send money to our family.” Come to think of it, COVID or no COVID, migrant workers are motivated primarily by the desire to support families back home. This time, however, the situation is compounded by the pandemic and fears over its life-changing repercussions.

Indeed, being away from one's family during a crisis can be unsettling to say the least. As pointed out by Dubey et al. (2020), “Remaining separated from family during an infectious disease outbreak may exact an enormous emotional toll” (p. 782). This is partly because the family stands as a source of emotional and psychological support under such circumstances, but the inability to be with them leads to debilitating anxiety. Nonetheless, it is also the very importance of family that motivates the migrant worker to confront the pandemic head-on and survive. Specifically, it is the need to protect the family's basic needs—generally, the chief reason for seeking employment overseas—which encourages the migrant to soldier on and remain healthy. Needless to say, this familial affection mitigates the distress brought about by the pandemic and helps bolster one's self-esteem (Yang et al., 2022).

### 4.3 Job precarity and stigmatization

For the migrant worker, another overwhelming fear is losing their means of livelihood and being deprived of the opportunity to support themselves and their loved ones financially. The worry is understandably greater if the worker and their family do not have any sense of financial security, and losing employment could reduce them to a hand-to-mouth existence. What compounded the situation was the prejudicial treatment accorded the lowly migrant by their employers and other locals who viewed them as transmitters of the disease.

In the case of the domestic helpers, the mobility restrictions, while necessary, compounded their daily chores. As the persona in “Pandemic” by Warmingsih (Kopi blogsite, 2020) observes, “Everyone is home, my workload already plenty,/now double/I struggle with the amount of task I have to juggle.” To make matters worse, the persona has to contend with distorted claims about the complicity of migrant workers in the spread of the contagion. Indeed, as the Slovenian philosopher Slavoj Zizek (2020) has argued, Covid-19 could be likened to an “external intruder” whose disruption of normality led to more conspiracy theories and increased racism—i.e., “blaming the ethnic Other for the outbreak” (p. 135). In a way, the pandemic reinforced the systemic inequalities and political conflicts lying beneath the surface. While it is true that, considering its scope, the global emergency foregrounded the need for collective action, it also reinforced old and unresolved injustices that have been victimizing marginal constituencies, among them migrant workers.

In “First Draft” by Sakir Khokan (2020), the persona does not mince words about the plight of migrants during the early lockdowns. S/He relates that when the order to observe physical distancing came, the migrants could not but feel provoked by the new measure primarily because of the physical limitations of their living space—“How to keep distance in this crammed space?” The succeeding lines sardonically imply how migrant workers' perennial concerns were not being addressed at the same time that physical distancing was being required—“Some measure the room with tape. Others measure the dimensions of wrinkles on their forehead.” There is also the seeming accusation of duplicity and hypocrisy on the part of authorities tasked to implement the measures that they themselves seem to transgress—“The administration has stated, wearing masks is mandatory/but they do not have masks.” This and related lines in the poem constitute a scathing criticism of ill-thought-out strategies to contain the pandemic which, unfortunately, does not take cognizance of their immediate and lasting effects on society's more vulnerable sectors—“If they don't have masks, how will they wear them/They gape at themselves. They cannot understand who is belittling whom, in this race of life.” One may also interpret the word “race” in this case as a reference to racial and ethnic tensions in which the migrant may find themselves embroiled.

There are also lines from the same poem that suggest the subtle and none- too-subtle forms of injustice that migrants struggle against, including their being “bound by (sic) the agent's fee,/their lives… mortgaged to the unknown.” And while the virus is expected to weaken over time, thanks to a massive vaccination campaign and other steps that have been taken to improve the situation, the persona bemoans the general indifference toward migrant workers—“Days and years pass,/the beautiful city changes, but not their salary.” There is also fear that the migrants will go back to their homeland under even worse circumstances—“They know the High Commission is on here/to parcel their corpses back home.” Still, it's not just government that the persona points an accusing finger at: Journalists, artists, and intellectuals who are either equivocating about the plight of migrants or downplaying their afflictions are also accountable. The words of Henry Giroux (2021) are instructive: the pandemic is “much more than a medical crisis… At its core, it is a political and ideological crisis” (p. xvi).

Nonetheless, it bears stressing that even prior to the pandemic, there had been widespread and systemic xenophobia targeting migrant workers in many developed countries. The pandemic made conditions worse for migrants because aside from discrimination, they also had to put up with the challenge posed by the disruption of remittance flow, thus driving them and their families back home to further hardship and deprivation. UNICEF (2020) noted that the lockdowns and quarantines increased the incidence of “abuse and exploitation and the trafficking of women migrant workers.”

### 4.4 Hope and resilience

Any crisis can understandably dampen the spirits of people, but robust hope should remain to serve as a cushion against the anxiety, depression, and restlessness that the pandemic has caused. It is a means by which individuals and groups maintain their stability in the midst of a formidable adversity; it is also what provides the courage and energy to keep on living. In other words, the appropriate attitude to face a crisis should be one of hope and, if need be, of militant optimism.

In “Humanity Love” by Haidee Roiles (Kopi blogsite, 2020), the persona hankers for the “olden (sic), cheerful days” before the pandemic, but sadness is quickly overturned by the expression of optimism—“We are the hope and the love for/humanity is the best weapon to face this/challenging situation. With so much love.” Note how the persona resists despair with a concern for humanity, mindful that the crisis would not last and completely blight the future, as the future could be different but not destroyed beyond recovery. As the philosopher Albert Camus (1956) wrote, “(I)n certain cases, carrying on, merely continuing, is superhuman” (p. 35).

The same message resonates with “Pandemic” by Warmingsih (Kopi blogsite, 2020). The persona criticizes the allegations made about/against migrant workers, but the persona does not dwell on bitterness, and instead chooses to celebrate the generous efforts to provide succor to fellow migrants dealing with their vulnerability in foreign territory—“I witness plenty of heroic deeds/Countless kind deeds helping migrant workers in need/Give me a glimpse of hope, a tiny light.” Indeed, well-meaning citizens in the territory extend assistance to others regardless of their status and origins especially at a time of great crisis affecting everybody; this suggests that while migrants can be on the receiving end of discrimination, there will always be individuals and groups whose action bespeaks authentic generosity and benevolence.

It bears emphasizing, however, that survival hinges primarily on oneself. This is not to undermine the responsibility of government or other institutions to ensure the welfare of people, but on an individual level, there is no other option but to be brave in the face of an existential threat. Showing weakness and vacillation can only make one even more susceptible. This is the message conveyed in some of the lines from the poem “The Pen is My Witness” by Joralyn Mounsel (Singapore Red Cross, 2021) where the persona comes to grips with the “dark isolations” and “illusions” generated, if not exacerbated, by the pandemic. The attitude is ascribed to a strong and unshakeable faith which has “brought (the persona) back to reality.” Indeed, for many people, faith served as the main refuge and source of emotional and mental strength to ward off the surrounding gloom of the times.

In the poem “What is Home, When the Heart is a Nomad?” by Zasim (*Call and Response 2*, 2021, pp. 72–75), there is a suggestion of optimism and confidence that better and brighter days are forthcoming. The feeling of hope is also expressed in the description—and renewed appreciation—of sights that would otherwise be taken for granted—“And the sun will shine at an angle/Birds will fly in the open sky/Slicing through the dark shadows again… The languor of despair will dissipate and the golden day will come.” What is glaring in the poem is the citing of seemingly opposed ideas pertaining to the pandemic—i.e., its adverse impact on people juxtaposed with the resilience and tenacity of the human spirit to meet it head-on.

Such expressions of hope should not be reduced to mere coping mechanisms in the midst of tragedy. As pointed out by Andrews (2014), “narrative and imagination are integrally tied to one another” (p. 1). Moreover, hope/imagination imbricates not just “our elevated thoughts about the world as it might be,” but also “the very minutiae of our daily lives” (p. 1). If there was any consolation, the crisis encouraged people to look inward and exercise self-care which had been rendered secondary to the more material concerns of modernity. As the persona reveals, the pandemic was a learning period for appreciating other people's “love…/with or without prejudices.”

## 5 Conclusion

In terms of stylistics and form, the poems of migrant workers may not be able to measure up to deeply entrenched literary conventions. Not a few of the sample pieces are riddled with grammatical errors and the vocabulary is sparse. Such observations, however, can be easily explained. For one, English (in which the poems are written) is not the first language of the migrant authors, except for one exception who was born and raised in the US. Secondly, most of the migrant authors are mostly engaged in skilled work and come from working class backgrounds without the trappings of middle-class or upper-class education. For many countries, proficiency in English is an indicator of a relatively privileged upbringing, though it is by no means a measure of intelligence.

It may be important to find out in future research how other art forms, say, those in the visual arts, differed from the poeticization of human suffering, loss, resilience, and hope during the pandemic. What can be factored in, for instance, is the sense of immediacy in poetry and other literary genres when published online. As shown in the paper, many of the poems that won in competitions and ended up in anthologies had originally appeared in digital form and, as such, could be easily accessed and appreciated, thus immediately providing the reader the opportunity to partake in the grief and/or optimism of the poem. The same thing cannot be said of other art forms whose expression of human frailty and hope is oftentimes made available only within the hallowed confines of the art gallery.

While it is true that the global health emergency, true enough, impacted the whole of humanity, it is no exaggeration that some sectors were impacted more terribly. The narratives of migrant workers, in whatever form, should therefore be recuperated in order to come up with a wholistic assessment of the situation at the height of the pandemic. Moreover, the well-trumpeted declarations of inclusivity in an increasingly globalizing world can only sound believable when turned into concrete steps that include retrieving the voices of marginal groups. Such an undertaking should necessarily involve reading works that may not measure up to conventional aesthetic standards but should be appreciated instead based on what they let us know about cultures and groups that exist on the fringes of current political, economic, cultural, and epistemic arrangements. More than those of dominant classes in any given society, it is the voices of these marginal groups that can provide the tools for challenging dominant institutions and authority—a critical approach remains and should always remain integral to any discourse of democratization.

## Data Availability

The datasets presented in this study can be found in online repositories. The names of the repository/repositories and accession number(s) can be found below: Amnesty International (2020). Singapore: Over 20,000 migrant workers in quarantine must be protected from mass infection. Available at: https://www.amnesty.org/en/latest/news/2020/04/singapore-migrant-workers-quarantine-protected-mass-infection/ (accessed July 15, 2024). Andrews, M. (2014). Narrative imagination and everyday life. New York, NY: Oxford University Press. Business and Human Rights Resource Center (2023). New database exposes business inaction on migrant worker abuse. Available at: https://www.business-humanrights.org/en/from-us/media-centre/new-database-exposes-business-inaction-on-migrant-worker-abuse/ (accessed August 23, 2024). Butler, J. (2021). Creating an Inhabitable World for Humans Means Dismantling Rigid Forms of Individuality. Available at: https://time.com/5953396/judith-butler-safe-world-individuality/ (accessed July 16, 2024). Camus, A. (1956). The fall (Trans. Justin O'Brien). New York, NY: Vintage Books. Cleofas, J. V., Eusebio, Ma. C. SC., and Pacudan, E. J. P. (2021). Anxious, Apart, and Attentive: A Qualitative Case Study of Overseas Filipino Workers' Families in the time of COVID-19. The Family Journal. doi: 10.1177/10664807211006339 Creative minds commemorate world mental health day (2021). Available at: https://redcross.sg/news-and-stories/10-events/1138-creative-minds-commemorate-world-mental-health-day.html (accessed August 22, 2024). Delgado Wise, R. (2018). Is there a space for counterhegemonic participation? Civil society in the global governance of migration? Globalizations, 15:6, 746–761, doi: 10.1080/14747731.2018.1484204 Delgado-Wise, R. (2014). A Critical Overview of Migration and Development: The Latin American Challenge. Annual Review of Sociology, 40:1, 643–663. doi: 10.1146/annurev-soc-071811-145459 Dubey, S., Biswas, P., Ghosh, R., Chatterjee, S., Dubey, M.J., Chatterjee, S., Lahiri, D., and Lavie, C. (2020). Psychosocial impact of COVID-19. Diabetes &amp; Metabolic Syndrome: Clinical Research &amp; Reviews, 14: 779–788. doi: 10.1016/j.dsx.2020.05.035 Giroux, H. (2011). Living in the Age of Imposed Amnesia: The eclipse of democratic formative culture. Policy Futures in Education, 9:5, 548–552. doi: 10.2304/pfie.2011.9.5 Giroux, H. (2021). Race, politics, and pandemic pedagogy: Education in a time of crisis. London, UK: Bloomsbury Academic. Haith, C. (2020). Pandemics from Homer to Stephen King: What we can learn from literary history. Available at: https://theconversation.com/pandemics-from-homer-to-stephen-king-what-we-can-learn-from-literary-history-133572 (accessed August 8, 2024). Helms, B. and Lebland, D. (2019). Global migration: Causes and consequences. Oxford research encyclopedia of politics. Available at: https://oxfordre.com/politics/view/10.1093/acrefore/9780190228637.001.0001/acrefore-9780190228637-e-631 (accessed July 8, 2024). Hill, R., Lakner, C., Mahler, D.G., Narayan, A., and Yonzan, N., (2021). Poverty, median incomes, and inequality in 2021: A diverging recovery (English). Washington, D.C.: World Bank Group. Available at: http://documents.worldbank.org/curated/en/936001635880885713/Poverty-Median-Incomes-and-Inequality-in-2021-A-Diverging-Recovery (accessed August 8, 2024). Illmer, A. (2020). Covid-19: Singapore migrant workers infections were three times higher. Available at: https://www.bbc.com/news/world-asia-55314862 (accessed August 18, 2024). Jin, J.Y. (2021). In lockdown for 18 months, Singapore's migrant workers yearn for freedom. Available at: https://www.nytimes.com/2021/09/29/world/in-lockdown-for-18-months-singapores-migrant-workers-yearn-for-freedom.html (accessed July 16, 2024). Jones, K., Mudaliar, S., and Piper, N. (2021). Locked down and in limbo: The global impact of COVID-19 on migrant worker rights and recruitment. Geneva, Switzerland: International Labor Organization. Available at: https://www.ilo.org/publications/locked-down-and-limbo-global-impact-covid-19-migrant-worker-rights-and (accessed June 26, 2024). Khokan, H., Navato, B., Han, P.Y., and Ip, J. (eds.) (2021). Call and response 2: A Singapore migrant anthology. Singapore: Math Paper Press. Khokan, Z.H. (2020). The plight and poetry of Singapore's migrant workers. Available at: https://artreview.com/the-poetry-of-singapore-migrant-workers/ (accessed June 14, 2024). Kopi blogsite (2020). Poetry From Migrant Workers Shines a Light on Their COVID-19 Plight. Available at: https://thekopi.co/2020/05/17/poetry-migrant-workers-covid-19/ (accessed July 23, 2024). Kotz, D. (2002). Globalization and neoliberalism. Rethinking Marxism: A Journal of Economics, Culture & Society, 14:2, 64–79. doi: 10.1080/089356902101242189 Mayor, C. and Pollack, S. (2022). Creative Writing and Decolonizing Intersectional Feminist Critical Reflexivity: Challenging Neoliberal, Gendered, White, Colonial Practice Norms in the COVID-19 Pandemic. Afflia. 37:3, 382–395. doi: 10.1177/08861099211066338 Migration Data Portal (2023). Migration data relevant for the COVID-19 pandemic. Available at: https://www.migrationdataportal.org/themes/migration-data-relevant-covid-19-pandemic (accessed August 23, 2024). Moratilla, N. C. (2019). Occluded histories: Philippine labor after EDSA. Philippine Political Science Journal, 4: 1-2, 3-31. doi: 10.1163/2165025X-12340001 Singapore Ministry of Manpower (2023). Foreign workforce numbers. Available at: https://www.mom.gov.sg/foreign-workforce-numbers (accessed May 25, 2024). Statista (2024). Total number of novel coronavirus (COVID-19) cases in the Asia-Pacific region as of April 2024, by country or territory. Available at: https://www.statista.com/statistics/1104263/apac-covid-19-cases-by-country/ (accessed May 28, 2024). The Independent Singapore (2023). Foreigner population grows 13.1 per cent as Singapore needs more workers. Available at: https://theindependent.sg/foreigner-population-grows-13-1-per-cent-as-singapore-needs-more-workers/ (accessed June 2, 2024). UNICEF (2020). End stigma and discrimination against migrant workers and their children during COVID-19 pandemic. Available at: https://www.unicef.org/eap/press-releases/end-stigma-and-discrimination-against-migrant-workers-and-their-children-during (accessed August 6, 2024). United Nations Network on Migration (2020). The impact of COVID-19 on family remittances: a lifeline cut for migrant families. Available at: https://migrationnetwork.un.org/resources/impact-covid-19-family-remittances-lifeline-cut-migrants-families (accessed May 23, 2024). United Nations Office on Drugs and Crime (2021). COVID-19 and the smuggling of migrants: A call for safeguarding the rights of smuggled migrants facing increased risks and vulnerabilities. Available at: https://www.unodc.org/documents/human-trafficking/SOM_and_COVID-19_Publication_final_EN_final.pdf (accessed August 8, 2024). World Bank (2023). Migrants, Refugees, and Societies: World Development Report. Available at: https://www.worldbank.org/en/publication/wdr2023 (accessed June 18, 2024). Yang C, Gao H, Li Y, Wang E, Wang N and Wang Q (2022) Analyzing the role of family support, coping strategies and social support in improving the mental health of students: Evidence from post COVID-19. Frontiers in Psychology, 13:1064898, 1-17. doi: 10.3389/fpsyg.2022.1064898 Zizek, S. (2020). Pandemic! 2: Chronicles of a time lost. London, UK: OR Books.
